# mTOR blockade prevents progressive proteinuria but induces hyperglycaemia in obese Dahl salt‐sensitive rats before puberty

**DOI:** 10.1113/EP092435

**Published:** 2025-10-27

**Authors:** Sautan Mandal, Andrea K. Brown, Ubong S. Ekperikpe, Anukool A. Bhopatkar, Krystle M. Hughes, Tyler D. Johnson, Jonita S. Cooper, Konnor T. Wilbert, Denise C. Cornelius, Jan M. Williams

**Affiliations:** ^1^ Department of Pharmacology and Toxicology University of Mississippi Medical Center Jackson Mississippi USA

**Keywords:** prepubertal/childhood obesity, rapamycin, renal injury, salt‐sensitive rat, SS^LepR^mutant rat

## Abstract

Previous studies have demonstrated that mammalian target of rapamycin (mTOR) activity is significantly increased in the kidneys of Dahl salt‐sensitive (SS) rats during the development of renal injury. Therefore, in the present study we examined whether blockade of mTOR with rapamycin inhibits renal injury in Dahl salt‐sensitive leptin receptor mutant (SS^LepR^mutant) rats. Four‐week‐old SS and SS^LepR^mutant rats were treated with either vehicle (saline, i.p.) or rapamycin (1.5 mg/kg/day, i.p.) for 4 weeks. Body weight was significantly higher in SS^LepR^mutant rats than in SS rats, and chronic treatment with rapamycin significantly decreased body weight only in SS^LepR^mutant rats. In vehicle‐treated rats, blood glucose levels were within the physiological range (≤120 mg/dL), and rapamycin treatment induced hyperglycaemia in SS^LepR^mutant rats (550 ± 54 mg/dL). Additionally, plasma insulin was significantly increased in SS^LepR^mutant rats versus SS rats, and rapamycin reduced plasma insulin in SS^LepR^mutant rats. Proteinuria was significantly higher in SS^LepR^mutant rats versus SS rats (564 ± 104 and 26 ± 12 mg/day, respectively), and rapamycin significantly decreased proteinuria in SS^LepR^mutant rats (48 ± 18 mg/day). Moreover, SS^LepR^mutant rats displayed renal hyperfiltration and marked glomerular and tubular injury compared with SS rats, and rapamycin improved these renal abnormalities in SS^LepR^mutant rats. Interestingly, we observed that renal sodium glucose cotransporter 2 (SGLT2) expression was significantly elevated in SS^LepR^mutant rats versus SS rats, and rapamycin markedly reduced renal SGLT2 expression in SS^LepR^mutant rats. Overall, these data indicate that mTOR plays an important role in renal metabolic disease in obese SS^LepR^mutant rats before puberty and suggest that rapamycin might prevent renal hyperfiltration associated with obesity by decreasing renal SGLT2 activity.

## Abstract (highlights)

- **What is the central question of this study?**
  Does inhibition of mammalian target of rapamycin (mTOR) with rapamycin prevent the early development of renal injury in prepubertal SS^LepR^mutant rats with obesity, and what mechanisms mediate these effects?
- **What is the main finding and its importance?**
  Our study shows that hyperinsulinaemia‐driven mTORC1 activation and SGLT2 upregulation contribute to early renal injury in obesity, independent of hyperglycaemia. Rapamycin reduced insulin levels, downregulated SGLT2, lessened renal injury and increased anti‐inflammatory cytokines (interleukin‐4 and interleukin‐10). These findings highlight mTORC1 inhibition as a potential early intervention for obesity‐related renal disease in children, moving beyond traditional blood pressure and glucose control.

## 1 INTRODUCTION

Childhood/prepubertal obesity is a major public health concern and growing epidemic in the USA. Clinical studies have shown that prepubertal obesity is associated with microalbuminuria, hyperinsulinaemia and increased risk for renal injury (Carullo et al., 2023; Kaneko et al., 2011). Early renal injury during prepubertal obesity increases the risk of developing chronic kidney disease and cardiovascular diseases later in life (Carullo et al., 2023). Obesity‐induced renal disease during prepubertal obesity can also occur in the absence of diabetes and hypertension (García‐Carro et al., 2021). However, there are a limited number of studies focused on obesity‐induced renal disease, and the mechanisms are unclear.

Insulin is a key anabolic hormone that plays a vital role in glucose homeostasis. In the early stages of type 2 diabetes, hyperinsulinaemia and insulin resistance (IR) contribute to renal injury through two primary mechanisms. First, hyperinsulinaemia increases renal glucose reabsorption, which also causes sodium retention, leading to elevated glomerular filtration rate (GFR) and renal injury (Naderpoor et al., 2017; Pereira‐Moreira & Muscelli, 2020; Whaley‐Connell & Sowers, 2017). Second, IR triggers the release of pro‐inflammatory cytokines, causing endothelial dysfunction and injury to glomeruli and tubules (Chen et al., 2015; Rehman & Akash, 2016; Takeda et al., 2020; Wieser et al., 2013). Moreover, insulin regulates several intracellular downstream pathways, including the mammalian target of rapamycin complex 1 (mTORC1) (Walker et al., 2019; Yoon, 2017). Insulin stimulates protein and lipid synthesis within cells by activating mTORC1, a key regulator of cell growth and proliferation (Han & Wang, 2017; Yoon, 2017). Previous studies have demonstrated that activated mTOR signalling promotes renal fibrosis, acute kidney injury and glomerular injury (Gui & Dai, 2020; Jiang et al., 2013; Li et al., 2015). In high‐fat‐fed obese rats, IR is linked to overactivation of the mTOR pathway, which also disrupts insulin signalling and promotes renal injury (Sun et al., 2019). Recent studies have demonstrated a link between mTORC1 activation and renal injury in SS rats. Kumar et al. (2020, 2017) found that a high‐salt diet activates the mTORC1 pathway in the kidneys of SS rats, resulting in urinary albumin excretion. Likewise, Shimada et al. (2022) found that increased renal perfusion pressure activates mTORC1, leading to renal immune cell infiltration and inflammation in SS rats. Notably, treatment with rapamycin, an mTORC1 inhibitor, reduced immune cell infiltration, lowered blood pressure and lessened renal injury in both studies (Kumar et al., 2020, 2017; Shimada et al., 2022). However, the role of upregulated mTORC1 signalling in the early progression of renal injury during obesity has not been studied before. SS^LepR^mutant rats are a well‐characterized model to study renal injury associated with obesity prior to puberty (Ekperikpe, Mandal et al., 2023; Ekperikpe, Poudel et al., 2023; Poudel et al., 2022). We previously observed that the development of progressive renal injury in obese SS^LepR^mutant rats is associated with elevated renal mTORC1 expression before puberty. Therefore, the present study focused on whether blockade of mTORC1 by rapamycin inhibits the early progression of renal disease in SS^LepR^mutant rats prior to puberty.

## 2 MATERIALS AND METHODS

### 2.1 Ethical approval

In the present study, we used Dahl salt‐sensitive (SS) and Dahl SS leptin receptor (SS^LepR^mutant) mutant male and female prepubertal rats at 4 weeks of age. Rats were obtained from our in‐house colony of heterozygous SS^LepR^mutant rats, which were originally created at the Medical College of Wisconsin by zinc finger nuclease technology (McPherson et al., 2016). Genotyping of SS and SS^LepR^mutant rats was done by the Molecular and Genomic core facility at University of Mississippi Medical Centre (UMMC). Throughout the study, rats were housed with a 12 h light–12 h dark cycle, maintained at room temperature and provided with free access to food and water. The rats were fed a chow diet containing 0.6% NaCl (TD8640; Envigo, Madison, WI, USA) and kept in the Laboratory Animal Facility of the UMMC [Animal Welfare Assurance Number D16‐00174 (A3275‐01)], which is approved by the American Association for the Accreditation of Laboratory Animal Care [AAALAC, UMMC AAALAC accreditation (12 July 2024)]. All protocols were approved by the University of Mississippi Medical Centre Institutional Animal Care and Use Committee. Rapamycin was purchased from LC laboratories (Catalogue‐R‐5000, Woburn, MA, USA).

### 2.2 Protocol

Four‐week‐old SS and SS^LepR^mutant rats were used for the present study. At baseline, rats were weighed, and non‐fasting blood glucose levels were measured from tail vein blood (via glucometer from AimStrip Plus, San Antonio, TX, USA). Then they were placed in metabolic cages overnight to collect urine to measure proteinuria via the Bradford method (Bio‐Rad Laboratories, Hercules, CA, USA), as described previously (Ekperikpe, Poudel et al., 2023; Poudel et al., 2020). After baseline measurements, rats were divided and randomly assigned into four groups: (1) SS and (2) SS^LepR^mutant rats treated with vehicle (saline, i.p., daily), (3) SS and (4) SS^LepR^mutant rats treated with rapamycin (1.5 mg/kg/day, i.p.; LC laboratories, Catalog‐R‐5000, Woburn, MA, USA) for 28 days. The dose of rapamycin was selected based on previous reports (Bridle et al., 2009; Kumar et al., 2017; Shimada et al., 2022). Every 2 weeks rats were placed in metabolic cages and the body weight, non‐fasting blood glucose and proteinuria were measured. At the end of the protocol, the plasma and urine collected were used to estimate GFR via creatinine clearance (Catalogue‐DICT‐500, Bioassay Systems, Hayward, CA, USA). According to the manufacturer's instructions, plasma and urine creatinine concentrations were measured. Urine and plasma concentrations were normalized to urine flow rate and kidney weight. Kidney injury molecule‐1 (KIM‐1) excretion rate was measured (Abcam, Waltham, MA, USA) in the urine and normalized to urine flow rate according to the manufacturer's instructions. We have measured the urinary sodium and glucose excretion with a Vet Axcel Chemistry Analyzer, which is also normalized to urine flow rate.

Two days before harvest, rats were anaesthetized, and a sterile chronic catheter was placed in the carotid artery for the measurement of mean arterial blood pressure (MAP). After 1 day of recovery, catheters were connected to pressure transducers (MLT0699, AD Instruments, Colorado Springs, CO, USA), and MAP was measured as described previously (Ekperikpe, Poudel et al., 2023). Male and female animals were combined in the analysis based on prior data showing no significant differences in MAP between sexes in SS and SS^LepR^mutant rats (Poudel et al., 2022).

On the day of harvest, rats were placed in an induction chamber with 3% isoflurane (Covetrus, Dublin, OH, USA) delivered at an oxygen (O_2_) flow rate of 1.5–2 L/min to induce general anaesthesia. Once anaesthetized, they were transferred to a heating pad‐equipped surgical table to maintain body temperature. During the surgical procedure, anaesthesia was maintained with 2%–2.5% isoflurane and 1.5–2 L/min of O_2_ administered via a nose cone. Then, a midline incision was made to expose the abdominal cavity. A blood collection needle (BD Vacutainer, Franklin Lakes, NJ, USA) was inserted into the abdominal aorta, and blood was collected for the measurement of plasma insulin (Mercodia rat insulin ELISA, Uppsala, Sweden) and triglyceride (Cayman Chemical, Ann Arbor, MI, USA). The kidneys were perfused using 20 mL of normal saline. The pancreas and kidney were harvested and weighed. One kidney was stored in 10% formalin and embedded in paraffin for histology and immunohistochemistry, and the other one was snap‐frozen in liquid nitrogen and stored at −80°C. Renal cytokines were measured using a Bio‐Plex Pro Rat Cytokine 3‐Plex Assay Reagent Kit on a Bio‐Rad Bio‐Plex 200 system as described by the manufacturer's protocol (Bio‐Rad Laboratories). The cytokines that were measured were interleukin (IL)‐4 and IL‐10. Renal mTORC1 levels were measured by ELISA (catalogue no. MBS1602979, MyBioSource, San Diego, CA, USA) from the frozen left kidney.

### 2.3 Renal and pancreatic histology

The kidney and pancreas were embedded into paraffin and cut into 5‐µm‐thick sections. Kidney sections were stained with Periodic Acid–Schiff and Picrosirius Red (PR). As described previously, Periodic Acid–Schiff‐stained tissue samples were scored on a scale ranging from zero to four to measure glomerular injury, where zero represents a normal glomerulus, one represents a 25% loss, two represents a 50% loss, three represents a 75% loss, and four represents a >75% loss of capillaries in the glomerular tuft (Ekperikpe, Poudel et al., 2023; Poudel et al., 2023). The PR‐stained tissue samples were used to measure renal fibrosis. Ten representative images from each sample were taken with an SeBa microscope equipped with a colour camera (Laxco Inc., North Creek, WA, USA). We analysed the percentage of each image stained red (collagen) by identifying the animal whose kidney had the most collagen. This was used to set a threshold parameter for red staining in the sections using NIS‐Elements D 3.0 software (McPherson et al., 2016; Spires et al., 2018). Thereafter, those same thresholding parameters were used for the red staining on each kidney image per rat in the study to measure renal fibrosis. Images were analysed using NIS‐Elements D 3.0 software.

Pancreatic sections were stained with Haematoxylin and Eosin for histological analysis. Six islet pictures were captured from each sample and used to measure islet size and the number of islets by ImageJ software (NIH, Stapleton, NY, USA). Islet size was measured by analysis of the area of each islet. Average values were normalized to total lobular area. Islet numbers were counted in each lobule. The average value of the number of islets was normalized to total lobular area.

### 2.4 Western blot

Kidney tissues containing both cortex and medulla were used for immunoblotting. Tissue samples were resuspended in lysis buffer cocktail, containing RIPA buffer (Sigma, St Louis, MO, USA) and protease inhibitor (Sigma). Tissue samples were homogenized, sonicated, and centrifuged at 5000 rpm for 5 min at 4°C. The supernatant was collected and centrifuged again at 11,180 g for 15 min at 4°C. Supernatants were collected and stored at −80°C until further use. Samples were prepared according to the standard western blot protocol (Bio‐Rad, USA). Next, we performed gel electrophoresis using a Bio‐rad Powerpac HC system. Then proteins were transferred from the gel to a polyvinylidene fluoride (methanol‐activated) membrane in a cold room for 1 h at 100 V. The membrane was incubated with blocking buffer (37571, Thermo Fisher, Waltham, MA, USA) for 1 h, followed by incubation overnight at 4°C with mouse monoclonal SGLT2 antibody (sc‐393350, SantaCruz, Dallas, TX, USA) and GAPDH antibody (sc‐47724, SantaCruz) at 1:1000 dilution. Next day, the membrane was washed in Tris‐buffered saline with Tween 20 (four times) and incubated with anti‐mouse horseradish peroxidase‐conjugated secondary antibody (Bio‐Rad, USA) at 1:8000 dilution for 1 h at room temperature in a 2D shaker. After that, the membrane was washed with Tris‐buffered saline with Tween 20 (four times) and Tris‐buffered saline (once). Chemiluminescence was performed to visualize the membrane using SuperSignal West Dura substrate (Thermo Fisher). Bands were imaged and analysed using ChemiDoc XRS^+^ imager (Bio‐Rad, USA) and ImageJ software (NIH, Stapleton, NY, USA) respectively.

### 2.5 Immunohistochemistry

Paraffin‐embedded kidney tissue slides were heated at 60°C for 30 min in a hot air oven. To remove excess paraffin, the slides were washed with a series of decreasing concentrations of xylene and increasing concentrations of ethyl alcohol, followed by heat‐induced antigen epitope retrieval in a sodium citrate buffer (pH 6.0) using a Coplin jar. After antigen retrieval, the tissues were blocked with 10% mouse serum for 2 h at room temperature. The blocked tissue slides were then incubated with mouse SGLT2 Alexa Fluor 488 antibody at a dilution of 1:100 (Santa Cruz) overnight at 4°C. The following day, the slides were washed three times with 1× Tris‐buffered saline to remove excess antibody. For quenching, 0.001% Evans Blue was applied. After quenching, the slides were mounted with 4′,6‐diamidino‐2‐phenylindole and visualized using a fluorescent microscope equipped with a Nikon imaging camera.

### 2.6 Statistical analysis

Data are expressed as the mean ± SD throughout the experiment results. The data were analysed and figures were made by GraphPad Prism 8 (GraphPad Software, San Diego, CA, USA). The mean difference at a single time point was analysed using two‐way ANOVA, followed by the Holm–Sidak multiple comparisons test. Temporal changes in protein excretion were compared between and within strains in both control and rapamycin‐treated groups using three‐way ANOVA, followed by Tukey's multiple comparisons test. A value of *p *< 0.05 was considered as significantly different.

## 3 RESULTS

### 3.1 Renal protein expression of mTORC1 and metabolic parameters

The effects of rapamycin treatment on mTORC1 expression and metabolic parameters are represented in Table 1. Renal mTORC1 protein levels were significantly increased in SS^LepR^mutant rats compared with SS rats (3.05 ± 0.79 and 1.88 ± 0.43 ng/mg protein, respectively), and chronic treatment with rapamycin normalized renal mTORC1 expression in SS^LepR^mutant rats without having an effect in SS rats (1.66 ± 0.26 and 1.21 ± 0.24 ng/mg protein, respectively). Body weight was significantly increased in SS^LepR^mutant rats when compared with SS rats (313 ± 30 and 216 ± 35 g, respectively). Rapamycin treatment markedly reduced body weight in SS^LepR^mutant rats (209 ± 34 g) compared with vehicle‐treated rats. Blood glucose levels were within the normoglycaemic range (≤120 mg/dL) in both control SS and SS^LepR^mutant rats (111 ± 8 and 105 ± 13 mg/dL, respectively). However, we observed a 5‐fold increase in blood glucose levels in SS^LepR^mutant rats (550 ± 54 mg/dL) when treated with rapamycin. Similar to blood glucose levels, we did not detect any difference in urine volume between vehicle‐treated SS and SS^LepR^mutant rats. However, chronic rapamycin treatment significantly increased urine volume only in SS^LepR^mutant rats. Plasma insulin levels were 10‐fold higher in control SS^LepR^mutant rats versus the levels measured in SS rats (10.82 ± 3.46 vs. 0.90 ± 0.26 ng/mL, respectively). Rapamycin treatment significantly reduced plasma insulin levels in SS^LepR^mutant rats (0.68 ± 0.20 ng/mL). Plasma triglyceride levels were markedly increased in SS^LepR^mutant rats compared with SS rats (650 ± 316 and 79 ± 39 mg/dL, respectively), and rapamycin reduced triglyceride levels in SS^LepR^mutant rats by almost 50%, without having an effect in SS rats. Urinary glucose and sodium excretion were similar between vehicle SS and SS^LepR^mutant rats. After chronic rapamycin treatment, glucose and sodium excretion were significantly increased only in SS^LepR^mutant rats.

**TABLE 1:** Effects of rapamycin treatment on metabolic and cardiovascular parameters in Dahl salt‐sensitive (SS) rats and Dahl salt‐sensitive leptin receptor mutant (SS^LepR^mutant) rats.

Metabolic parameters	Control		Rapamycin	
	SS	SS^LepR^mutant	SS	SS^LepR^mutant
Total rats (males, females)	11 (6, 5)	10 (5, 5)	13 (6, 7)	14 (7, 7)
mTORC1, ng/mg protein	1.88 ± 0.43	3.05 ± 0.79^†^	1.21 ± 0.24	1.66 ± 0.26^#^
Body weight, g	216 ± 35	313 ± 30^†^	186 ± 24	209 ± 34^#^
Blood glucose, mg/dL	111 ± 8	105 ± 13	111 ± 13	550 ± 54^†^, ^#^
Urine volume, mL	22 ± 10	23 ± 11	18 ± 6	85 ± 11^†^, ^#^
Insulin, ng/mL	0.90 ± 0.26	10.82 ± 3.46^†^	0.59 ± 0.27	0.68 ± 0.20^#^
Triglycerides, mg/dL	79 ± 39	650 ± 316^†^	58 ± 25	344 ± 302^†^, ^#^
Glucose excretion, mg/day	15.96 ± 6.39	34.41 ± 52.99	32.62 ± 38.20	2207 ± 383.6^†^, ^#^
Sodium excretion, mmol/day	2.26 ± 0.55	2.46 ± 1.19	1.86 ± 0.51	4.75 ± 0.79^†^, ^#^

*Note*: Values are mean ± SD. A value of *p *< 0.05 was considered a significant difference.

^†^ Significant difference from the corresponding value in SS rats within the same treatment.

^#^ Significant difference from the corresponding value in vehicle‐treated rats within the same strain.

### 3.2 Pancreatic islet histology

**FIGURE 1:**
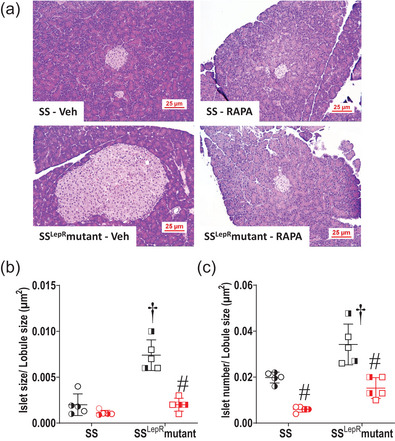
Representative images and analysis of pancreatic islets in Dahl salt‐sensitive (SS) and Dahl salt‐sensitive leptin receptor mutant (SS^LepR^mutant) rats treated with either vehicle or rapamycin for 4 weeks. Male and female rats are symbolized as open and partly filled symbols, respectively. (a) Haematoxylin and Eosin staining of pancreatic islets. (b) Islet size normalized by lobule size. (c) Number of islets normalized by lobule size. Values are the mean ± SD. A value of *p *< 0.05 was considered a significant difference. ^†^Significant difference from the corresponding value in SS rats within the same treatment. ^#^Significant difference from the corresponding value in vehicle‐treated rats within the same strain.

Representative images and corresponding analysis of pancreatic islet histology in control and rapamycin‐treated SS and SS^LepR^mutant rats are shown in Figure 1. Given that chronic rapamycin treatment induced hyperglycaemia and lowered plasma insulin levels (Table 1) in SS^LepR^mutant rats, we examined the effects of rapamycin on pancreatic islet histology. We observed an increase in pancreatic islet size and number in SS^LepR^mutant rats compared with SS rats (Figure 1). Chronic rapamycin administration significantly reduced both pancreatic islet size and number in SS^LepR^mutant rats compared with control animals.

### 3.3 Measurement of MAP and proteinuria

**FIGURE 2:**
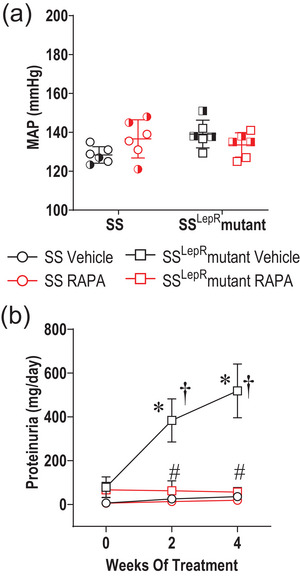
Effects of chronic treatment with rapamycin on mean arterial pressure (MAP) (a) and proteinuria (b) in Dahl salt‐sensitive (SS) and Dahl salt‐sensitive leptin receptor mutant (SS^LepR^mutant) rats. Male and female rats are symbolized as open and partly filled symbols, respectively. Values are mean ± SD. A value of *p *< 0.05 was considered a significant difference. ^*^Significant difference from the corresponding value within the same strain at baseline. ^†^Significant difference from the corresponding value in SS rats within the same treatment. ^#^Significant difference from the corresponding value in vehicle‐treated rats within the same strain.

The effects of chronic rapamycin treatment on MAP and proteinuria are shown in Figure 2. There was no significant difference in MAP among vehicle‐ and rapamycin‐treated SS and SS^LepR^mutant rats (Figure 2a). Proteinuria was similar at baseline between vehicle‐ and rapamycin‐treated SS and SS^LepR^mutant rats. At the end of study, proteinuria increased from 53 ± 19 to 564 ± 104 mg/day in vehicle‐treated SS^LepR^mutant rats, but only from 6 ± 6 to 26 ± 12 mg/day in vehicle‐treated SS rats (Figure 2b). However, rapamycin treatment reduced proteinuria only in SS^LepR^mutant rats, without affecting SS rats (56 ± 32 and 62 ± 26 mg/day, respectively).

### 3.4 Measurement of renal function and markers of renal injury

**FIGURE 3:**
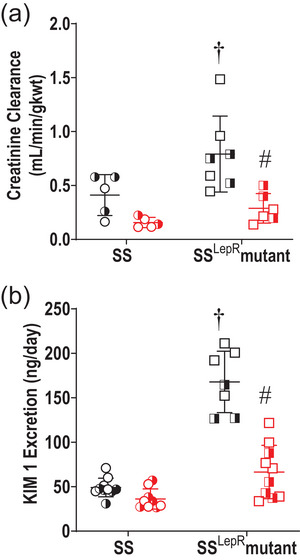
Effects of chronic treatment with rapamycin on creatinine clearance (a) and excretion of a marker of tubular injury, urinary kidney injury molecule‐1 (KIM 1) (b) in Dahl salt‐sensitive (SS) and Dahl salt‐sensitive leptin receptor mutant (SS^LepR^mutant) rats at 8 weeks of age. Male and female rats are symbolized as open and partly filled symbols, respectively. Values are the mean ± SD. A value of *p *< 0.05 was considered a significant difference. ^†^Significant difference from the corresponding value in SS rats within the same treatment. ^#^Significant difference from the corresponding value in vehicle‐treated rats within the same strain.

Effects of chronic rapamycin treatment on creatinine clearance and renal tubular injury are presented in Figure 3. We observed significant elevations in creatinine clearance (a measure of renal function and GFR) in vehicle‐treated SS^LepR^mutant versus SS rats, suggesting renal hyperfiltration (Figure 3a). Chronic rapamycin administration significantly reduced creatinine clearance in SS^LepR^mutant rats compared with vehicle‐treated rats. There was a 3‐fold increase in KIM‐1 excretion (a marker of renal tubular injury) in vehicle‐treated SS^LepR^mutant versus SS rats (Figure 3b), and rapamycin treatment significantly reduced KIM‐1 excretion in SS^LepR^mutant rats.

### 3.5 Renal histopathology

**FIGURE 4:**
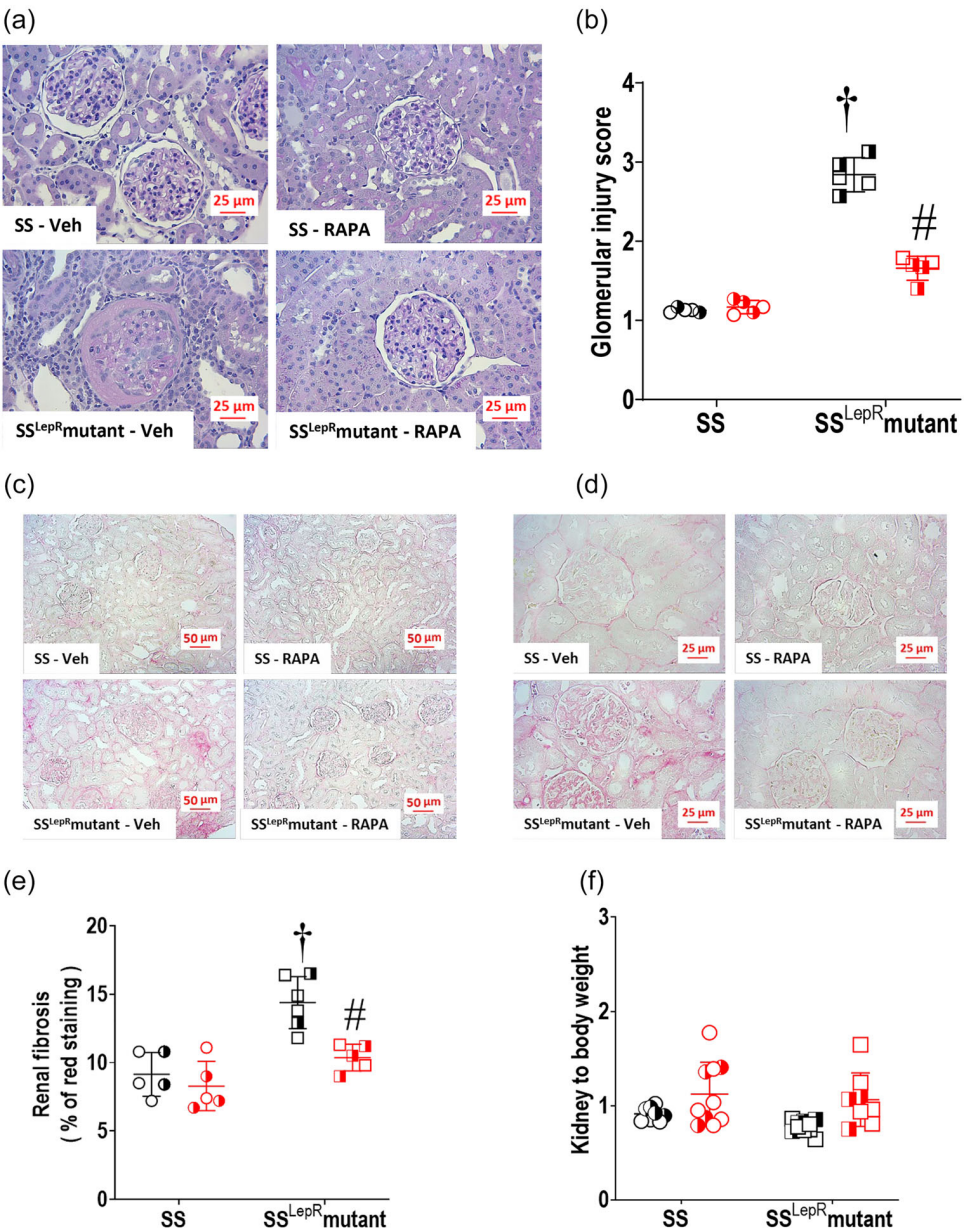
Representative images and analysis of renal histopathology in Dahl salt‐sensitive (SS) and Dahl salt‐sensitive leptin receptor mutant (SS^LepR^mutant) rats treated with either vehicle or rapamycin for 4 weeks. Male and female rats are symbolized as open and partly filled symbols, respectively. (a) Periodic Acid–Schiff staining for renal histology (×40 magnification). (b) Glomerular injury score. (c) Picrosirius red staining for renal fibrosis (×20 magnification). (d) Picrosirius red staining for renal fibrosis (×40 magnification). (e) Renal fibrosis measurement (percentage of red staining). (f) Kidney weight‐to‐body weight ratio. Values are the mean ± SD. A value of *p *< 0.05 was considered a significant difference. ^†^Significant difference from the corresponding value in SS rats within the same treatment. ^#^Significant difference from the corresponding value in vehicle‐treated rats within the same strain.

Representative images and corresponding analysis of renal histopathology in vehicle‐ and rapamycin‐treated SS and SS^LepR^mutant rats are presented in Figure 4. Kidneys from vehicle‐treated SS^LepR^mutant rats displayed increased mesangial expansion and glomerular injury in comparison to SS rats (Figure 4a,b). Administration of rapamycin significantly reduced glomerular injury in SS^LepR^mutant rats. We observed increased renal fibrosis in control SS^LepR^mutant versus SS rats (Figure 4c–e). Chronic rapamycin treatment significantly reduced glomerular injury and renal fibrosis in SS^LepR^mutant rats.

### 3.6 Renal cytokine measurement

**FIGURE 5:**
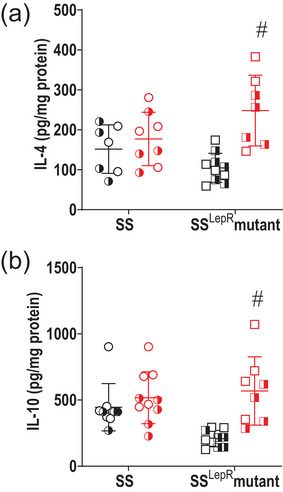
Effects of chronic rapamycin treatment on renal cytokines in Dahl salt‐sensitive (SS) and Dahl salt‐sensitive leptin receptor mutant (SS^LepR^mutant) rats at 8 weeks of age. Male and female rats are symbolized as open and partly filled symbols, respectively. The interleukin‐4 (IL‐4) (a) and interleukin‐10 (IL‐10) (b) cytokine levels in kidney; values are normalized by total kidney protein. Values are the mean ± SD. A value of *p *< 0.05 was considered a significant difference. ^†^Significant difference from the corresponding value in SS rats within the same treatment. ^#^Significant difference from the corresponding value in vehicle‐treated rats within the same strain.

The effects of chronic rapamycin treatment on renal cytokine levels in SS and SS^LepR^mutant rats are shown in Figure 5. We did not observe significant differences in renal IL‐4 and IL‐10 levels between SS and SS^LepR^mutant rats (Figure 5a,b). However, chronic rapamycin administration increased renal IL‐4 and IL‐10 levels in SS^LepR^mutant rats compared with vehicle‐treated SS^LepR^mutant rats, whereas no effect was seen in SS rats.

### 3.7 Immunohistochemistry and western blot analysis

**FIGURE 6:**
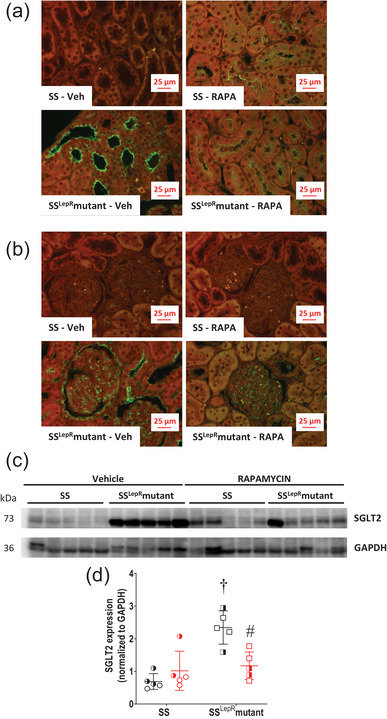
Effects of chronic rapamycin treatment on renal SGLT2 expression in Dahl salt‐sensitive (SS) and Dahl salt‐sensitive leptin receptor mutant (SS^LepR^mutant) rats at 8 weeks of age. Male and female rats are symbolized as open and partly filled symbols, respectively. (a) Renal tubular SGLT2 expression (green). (b) Glomerular SGLT2 expression (green). (c) Renal SGLT2 and GAPDH expression. (d) Western blot analysis of renal SGLT2 expression normalized by GAPDH. Values are the mean ± SD. A value of *p *< 0.05 was considered a significant difference. ^†^Significant difference from the corresponding value in SS rats within the same treatment. ^#^Significant difference from the corresponding value in vehicle‐treated rats within the same strain.

Renal expression of SGLT2 is shown in Figure 6. We observed increased expression of SGLT2 in the proximal tubules (Figure 6a) and glomeruli (Figure 6b) by immunohistochemistry in vehicle SS^LepR^mutant rats compared with SS rats. The upregulation of SGLT2 was confirmed by western blot analysis, which showed elevated SGLT2 protein levels in SS^LepR^mutant rats compared with SS rats (Figure 6c). Although SGLT2 is expressed primarily in proximal tubules, it has been also detected in podocytes and mesangial cells in diabetic conditions (Maki et al., 2019; Wakisaka et al., 2021). Chronic rapamycin administration significantly reduced renal SGLT2 expression in SS^LepR^mutant rats (Figure 6d).

## 4 DISCUSSION

Childhood obesity is a major public health concern in the USA (Cunningham et al., 2022; Sanyaolu et al., 2019). However, most obesity‐related renal disease research focuses on adults, with little effort in children. Moreover, recent studies suggest that overweight children have an increased risk of renal injury independent of hypertension and diabetes, with early signs of microalbuminuria (Correia‐Costa et al., 2018; Ding & Mak, 2015; Jadresic et al., 2019), but the mechanisms involved remain unclear. Recently, Cowley and colleagues observed that increases in mTORC1 activity play a major role in the development of hypertension‐induced renal injury and inflammation (Kumar et al., 2020, 2017; Shimada et al., 2022). Moreover, previous studies have demonstrated that increased mTOR signalling promotes renal fibrosis (Gui & Dai, 2020; L. Jiang et al., 2013). In the present study, the renal expression of mTORC1 was significantly higher in SS^LepR^mutant rats compared with SS rats prior to puberty. In SS^LepR^mutant rats, chronic treatment with the mTOR inhibitor, rapamycin, prevented progressive proteinuria, glomerular and tubular injury and renal hyperfiltration, effects that occurred independently of changes in arterial pressure. Additionally, the renal protective effects of rapamycin were accompanied by significant reductions in renal SGLT2 expression in SS^LepR^mutant rats. The most interesting finding was that chronic treatment with rapamycin reduced plasma insulin levels and stimulated hyperglycaemia. These findings suggest that increased mTORC1 signalling might contribute to the early onset of renal injury associated with prepubertal obesity in SS^LepR^mutant rats.

Consistent with our previous reports, we found no difference in MAP between vehicle‐treated SS and SS^LepR^mutant rats during this unique time period (Ekperikpe, Poudel et al., 2023; Poudel et al., 2023, 2020, 2022). Rapamycin treatment also did not affect arterial pressure in either strain. Although we hypothesized that rapamycin would reduce arterial pressure in SS^LepR^mutant rats, prior studies suggest that its effects are modulated by dietary salt intake. Kumar et al. (2017) reported that rapamycin increases arterial pressure in SS rats fed a low‐salt diet but decreased arterial pressure in those on a high‐salt diet. In the present study, both rat strains were maintained on a low‐salt diet, and no elevations in arterial pressure were observed. Interestingly, rapamycin stimulated hyperglycaemia in SS^LepR^mutant rats without an increase in arterial pressure, which was an unexpected finding, given previous reports from our group showing elevated arterial pressure in hyperglycaemic SS rats (Slaughter et al., 2013). There are two factors that might explain why we did not observe elevations in arterial pressure in both strains in response to rapamycin. First, our study used young, prepubertal rats, which might respond differently to rapamycin treatment. Second, in SS^LepR^mutant rats, rapamycin‐induced hyperglycaemia was accompanied by reduced renal expression of SGLT2, leading to increased sodium excretion and potentially preventing any rise in arterial pressure. These findings suggest that the arterial pressure response to rapamycin might depend on age and dietary salt intake.

Renal hyperfiltration is a hallmark of obesity‐induced renal disease (Basolo et al., 2023; Stefansson et al., 2016). However, whether obese children with early renal dysfunction exhibit elevated GFR remains unclear, largely owing to the lack of standardized and reliable methods for measuring GFR in paediatric populations (Jančič et al., 2022). Recent studies from our laboratory have shown that SS^LepR^mutant rats exhibit renal hyperfiltration during this early time period, measured by both creatinine and fluorescein isothiocyanate–sinistrin clearance (McPherson et al., 2020; Poudel et al., 2022). The underlying mechanisms, however, remain uncertain. Ekperikpe et al. (2023) demonstrated that reducing insulin levels or improving insulin sensitivity prevented elevations in GFR, suggesting that insulin might play a causal role in hyperfiltration in this model. Prior studies have shown that insulin enhances SGLT2 activity (Nakamura et al., 2015), which can impair the tubuloglomerular feedback response and increase GFR (Gérard et al., 2022). In the present study, elevated insulin levels in vehicle‐treated SS^LepR^mutant rats were associated with increased creatinine clearance and renal SGLT2 expression. Treatment with rapamycin reduced insulin levels, renal SGLT2 expression and GFR. These findings suggest that insulin‐stimulated SGLT2 expression might contribute to renal hyperfiltration in non‐diabetic, obese children with early renal disease.

One of the key findings from the present study is that rapamycin induced hyperglycaemia only in obese SS^LepR^mutant rats, but not in lean SS rats. This is consistent with prior studies showing that both reduced and augmented mTOR signalling can impair insulin action and contribute to hyperglycaemia (Houde et al., 2010; Larsen et al., 2006; Rodriguez‐Rodriguez et al., 2021). There are two potential mechanisms by which altered mTOR activity might promote hyperglycaemia. First, reduced mTOR signalling has been associated with a ‘starvation‐like diabetes’ phenotype, characterized by increased hepatic gluconeogenesis and ketogenesis, along with diminished insulin secretion from pancreatic β‐cells (Ardestani et al., 2018; Blagosklonny, 2012). Second, chronic mTORC1 overactivation can lead to insulin resistance by impairing glucose uptake in peripheral tissues (Khalid et al., 2021). Moreover, previous studies have shown that inhibitors of mTOR, such as rapamycin, induce insulin resistance in both rodents and humans (Lamming et al., 2012; Yilmaz et al., 2015). We hypothesize that in SS^LepR^mutant rats, elevated mTORC1 activity contributes to insulin resistance and that rapamycin‐induced inhibition of mTOR signalling stimulates hyperglycaemia. This is supported by our findings that rapamycin reduced the number and size of pancreatic β‐cells and decreased plasma insulin levels in SS^LepR^mutant rats, effects not observed in SS rats. One possible explanation is that SS^LepR^mutant rats are already insulin resistant, whereas SS rats maintain relatively normal insulin sensitivity during this unique time period. These results suggest that chronic rapamycin treatment might worsen insulin resistance and induce hyperglycaemia in obese individuals.

Surprisingly, inhibition of mTORC1 with rapamycin reduced progressive renal injury in hyperglycaemic SS^LepR^mutant rats. This finding was unexpected, given our previous work demonstrating that SS rats are highly susceptible to diabetes‐induced renal injury following streptozotocin treatment (Slaughter et al., 2013). Based on these earlier findings, one might have anticipated that rapamycin‐induced hyperglycaemia would exacerbate renal injury in SS^LepR^mutant rats. We hypothesize that the protective effect of rapamycin is mediated by the prevention of renal hyperfiltration, a hallmark of obesity‐related renal disease that contributes to glomerular and tubular injury, proteinuria, inflammation and fibrosis (Basolo et al., 2023; Stefansson et al., 2016). These pathological features were evident in vehicle‐treated SS^LepR^mutant rats. In support of this hypothesis, Cowley and colleagues recently demonstrated that increased renal perfusion pressure causes overactivation of mTORC1 (Shimada et al., 2022), and chronic blockade of mTORC1 by rapamycin slows the progression of renal injury in hypertensive SS rats (Kumar et al., 2019). Although the exact mechanism by which rapamycin prevents hyperfiltration remains unclear, two potential explanations exist. First, rapamycin reduces plasma insulin levels, and prior studies have shown that hyperinsulinaemia can elevate GFR (Di Bonito et al., 2014; Magen et al., 2022; Ricotti et al., 2018). Second, rapamycin suppresses renal SGLT2 expression, which might preserve tubuloglomerular feedback and limit GFR elevation (Chagnac et al., 2019; Vallon & Thomson, 2020). Moreover, insulin has also been reported to enhance SGLT2 activity in the kidney directly, further linking hyperinsulinaemia to renal hyperfiltration (Nakamura et al., 2015). Together, these findings suggest that rapamycin might protect against obesity‐related renal injury not by lowering blood glucose, but by disrupting the insulin–SGLT2–hyperfiltration axis.

Recent studies from our laboratory have shown that increased GFR contributes to renal inflammation and renal disease in SS^LepR^mutant rats prior to puberty (Ekperikpe, Poudel et al., 2023; McPherson et al., 2016; Poudel et al., 2022). Additionally, inhibition of inflammation slows the progression of proteinuria and renal injury in SS^LepR^mutant rats (Ekperikpe, Poudel et al., 2023; Poudel et al., 2023). In the present study, treatment with rapamycin, which is considered an immunosuppressive drug, inhibited proteinuria and renal injury in young obese SS^LepR^mutant rats by preventing renal hyperfiltration and increasing the renal anti‐inflammatory cytokines, IL‐4 and IL‐10. These cytokines are known to suppress pro‐inflammatory responses and promote tissue repair, which have been shown to prevent glomerular injury and to preserve renal function in models of ischaemic and immune‐mediated renal injury (Jung et al., 2012; Tipping et al., 1997). Overall, these findings suggest that rapamycin protects against renal injury in young obese SS^LepR^mutant rats by reducing hyperfiltration and promoting anti‐inflammatory cytokines.

Taken together, the findings from the present study indicate that young obese SS^LepR^mutant rats exhibit hyperinsulinaemia, renal hyperfiltration, elevated SGLT2 expression and inflammation, which are factors that contribute to early glomerular and tubular injury. These data support a model in which hyperinsulinaemia and hyperfiltration drive mTORC1 activation, promoting renal damage during the prepubertal period. Chronic rapamycin treatment attenuates this process by normalizing mTOR activity, reducing plasma insulin levels and downregulating renal SGLT2 expression. As a result, rapamycin effectively decreases renal hyperfiltration and inhibits renal injury in this model of obesity‐associated renal disease. Importantly, in addition to its metabolic effects, rapamycin also exhibits anti‐inflammatory properties, as evidenced by increased renal expression of the anti‐inflammatory cytokines IL‐4 and IL‐10 in treated SS^LepR^mutant rats. These findings suggest that mTORC1 inhibition might offer a multifaceted therapeutic strategy to prevent or delay the onset of renal injury in obese children at risk for early renal disease, albeit in combination with anti‐glycaemic drugs.

### 4.1 Clinical significance and perspective

The early onset of renal injury in obese children is currently a growing clinical concern, particularly as rates of paediatric obesity continue to rise. Current treatment approaches primarily target arterial pressure and glycaemic control in adults, with limited attention given to early intervention in children. Our findings suggest that hyperinsulinaemia‐induced activation of mTORC1 and increased SGLT2 expression might contribute to early renal injury in obesity, independent of hyperglycaemia. Rapamycin attenuated these effects by reducing insulin levels, downregulating SGLT2 and decreasing markers of glomerular and tubular injury. This highlights a potential therapeutic role for mTORC1 inhibition in early‐stage renal disease, particularly in the context of hyperinsulinaemia‐driven renal dysfunction. Moreover, the observed anti‐inflammatory effects of rapamycin, including increased renal IL‐4 and IL‐10, further underscore its promise as a new therapeutic strategy. These findings suggest that future studies are needed to evaluate the efficacy of mTORC1‐targeted therapies in paediatric populations at risk for obesity‐related renal disease.

## AUTHOR CONTRIBUTIONS

Sautan Mandal, Andrea K. Brown, Ubong S. Ekperikpe and Jan M. Williams conceived and designed research; Sautan Mandal, Andrea K. Brown, Ubong S. Ekperikpe, Anukool A. Bhopatkar, Krystle M. Hughes, Tyler D. Johnson, Jonita S. Cooper, Konnor T. Wilbert, Denise C. Cornelius and Jan M. Williams performed experiments; Sautan Mandal, Denise C. Cornelius and Jan M. Williams analysed data; Sautan Mandal, Anukool A. Bhopatkar, Krystle M. Hughes, Denise C. Cornelius and Jan M. Williams interpreted results of experiments; Sautan Mandal and Jan M. Williams prepared figures; Sautan Mandal and Jan M. Williams drafted the manuscript; Sautan Mandal, Anukool A. Bhopatkar, Krystle M. Hughes and Jan M. Williams edited and revised the manuscript; Sautan Mandal, Andrea K. Brown, Ubong S. Ekperikpe, Anukool A. Bhopatkar, Tyler D. Johnson, Krystle M. Hughes, Jonita S. Cooper, Konnor T. Wilbert, Denise C. Corneliu, and Jan M. Williams approved the final version of the manuscript and agree to be accountable for all aspects of the work in ensuring that questions related to the accuracy or integrity of any part of the work are appropriately investigated and resolved. All persons designated as authors qualify for authorship, and all those who qualify for authorship are listed.

## CONFLICT OF INTEREST

None declared.

## DISCLAIMERS

The content is solely the responsibility of the authors and does not necessarily represent the official views of the National Institutes of Health or American Heart Association.

## Data Availability

Data will be made available upon reasonable request.
